# 236 A pilot and evaluation of a dance intervention for young women following cancer treatment: Dance Exercise Intervention South East (The DEISE project)

**DOI:** 10.1093/eurpub/ckae114.143

**Published:** 2024-09-26

**Authors:** Martina Gooney, Patricia Hunt, Evan Matthews, Tracy McDaid, Michael O’Brien, Patricia Sheehan

**Affiliations:** South East Technological University, Waterford, Ireland; South East Technological University, Waterford, Ireland; South East Technological University, Waterford, Ireland; Solas Cancer Support Centre, Waterford, Ireland; Solas Cancer Support Centre, Waterford, Ireland; South East Technological University, Waterford, Ireland

## Abstract

**Purpose:**

There is emergent and strong evidence for effectiveness of dance-based physical activity for young women post cancer treatment. However, there is a need for knowledge on such physical activity implementation in a community setting. The aim of this project was to design, pilot and evaluate a 10-week, community-based peer-supported dance programme for young women at least 6 weeks post treatment for primary lymphoma, leukemia or breast cancer diagnosis. This programme focused largely on aerobic physical activity, functional fitness and social connection through supervised dance.

**Methods:**

In addition to quantitative indicators, qualitative descriptive research framed by the RE-AIM framework were used. Reflexive thematic analysis were used to analyse data collected in semi-structured interviews.

**Results:**

A sample of N = 7 women with experience of cancer and N = 2 peers participated in the qualitative research evaluation. Four overarching themes were explored: 1. The complex influences on commencing the dance class programme, 2. Participants’ perspective on the personal impact of the dance classes, 3. Interpersonal connections through the dance classes; more than exercise, and 4. Meaningful components within the dance programme.

**Conclusions:**

The findings highlighted the importance of addressing concerns about abilities to exercise, the impact of the social environment, developing confidence through the programme, and the perceived physical and mental health benefits. In the context of robust evidence on the health impacts of socially based physical activity for young women with experience of cancer, these findings offer implementation and feasibility knowledge for community based cancer support services.

**Funding:**

This project is funded by the National Cancer Control Programme (NCCP).

